# Male Infertility and Neurodegenerative Diseases: A Systematic Review of Associations and Molecular Mechanisms

**DOI:** 10.3390/ijms27073222

**Published:** 2026-04-02

**Authors:** Noora Jatan, Mustafa Al-Mashhadani, Skylar Dsouza, Sara Khan, Jonathan Mokhtar, Rachid Kaddoura, Stefan S. du Plessis

**Affiliations:** 1 College of Medicine, Mohammed Bin Rashid University of Medicine and Health Sciences (MBRU), Dubai Health, Dubai P.O. Box 505055, United Arab Emirates; noora.jatan@students.mbru.ac.ae (N.J.); mustafa.almashhadani@students.mbru.ac.ae (M.A.-M.); skylar.dsouza@students.mbru.ac.ae (S.D.); sara.khan@students.mbru.ac.ae (S.K.); jonathan.mokhtar@students.mbru.ac.ae (J.M.); 2 Research and Graduate Studies, Mohammed Bin Rashid University of Medicine and Health Sciences (MBRU), Dubai Health, Dubai P.O. Box 505055, United Arab Emirates

**Keywords:** male infertility, neurodegenerative disease, mitochondrial dysfunction, oxidative stress, proteostasis, systematic review

## Abstract

Male infertility has been viewed as a potential biomarker for systemic health and a predictor of future disease. With the global burden of neurodegenerative diseases (NDDs) on the rise, the current systematic review aims to synthesize the reported associations, risks, and shared molecular mechanisms between male infertility and NDDs. Following PRISMA 2020 guidelines, we systematically searched PubMed, Embase, Scopus, and Cochrane CENTRAL in January 2026. Studies examining the relationship between male infertility and NDDs were included. Screening and data extraction were performed by two independent reviewers with a third to resolve conflicts while quality appraisal (ROBINS-E and OHAT) was performed for all included studies. Studies used heterogeneous definitions of male infertility, including clinical diagnosis, semen parameters, and reproductive outcomes. Of the 1566 identified studies, 13 were included in this review including case-control studies, experimental investigations, in vitro studies, bioinformatic analyses, and pedigree studies. The available literature suggests possible mechanistic overlap between male infertility and neurodegenerative disease pathways, particularly in mitochondrial dysfunction, oxidative stress, and proteostasis. However, the evidence remains heterogeneous and preliminary, and large prospective studies are needed before male infertility assessment can be considered a marker of neurodegenerative risk. Registration: The study protocol was registered on PROSPERO (registration code: CRD420261301509).

## 1. Introduction

Male infertility, long considered a condition confined to the reproductive system, may be a potential barometer of male’s overall health and an indicator of future disease [1,2]. Affecting an estimated 56 million males globally, it has implications that extend far beyond procreation, such as being a potential marker for earlier mortality [3]. A growing body of evidence demonstrates that male infertility is a significant risk marker for a spectrum of systemic illnesses [4,5]. For instance, landmark studies have revealed that infertile males face a 37% higher risk of all-cause mortality, alongside significantly elevated risks for developing various cancers, diabetes, and major cardiovascular events [6,7,8,9]. This shift reframes male infertility not as an isolated issue, but as a critical, early-life marker of underlying systemic vulnerability.

Concurrently, the global burden of neurodegenerative diseases (NDD) continues to rise [10]. For the purposes of this review, NDDs include Alzheimer’s disease (AD), Parkinson’s disease (PD), Huntington’s disease (HD), amyotrophic lateral sclerosis (ALS), mitochondrial neurodegenerative syndromes, frontotemporal dementia, Lewy body dementia, and/or other clinically defined dementias/neurodegenerative disorders as reported. Among these, AD and related dementias affect over 55 million people globally, with prevalence projections reaching 139 million cases by 2050, representing a major driver of this burden [11]. This rapid escalation, driven largely by population aging, highlights the urgent need to identify early-life risk factors and predictive biomarkers to enable timely interventions and improved risk stratification. In parallel, growing evidence linking male infertility to a range of chronic systemic diseases has broadened investigative efforts, giving rise to hypotheses that male reproductive dysfunction may be associated with an increased risk of late-life neurodegeneration [12,13,14]. Emerging evidence suggests that alterations in cellular energy regulation, stress response pathways, and protein quality control systems may serve as mechanistic links between infertility and NDD. Despite the biological plausibility of these associations, epidemiological evidence linking male infertility phenotypes to the risk, incidence, or progression of specific NDDs remains limited and fragmented. Prior studies are frequently constrained by heterogeneous study designs, inconsistent definitions of infertility (ranging from clinical diagnoses to semen parameters or hormonal profiles), and variable approaches to neurological outcome ascertainment. To our knowledge, no prior review has systematically synthesized molecular and clinical evidence linking Male infertility to NDDs. Accordingly, we conducted a systematic review to synthesize the available evidence, evaluate the strength and consistency of reported associations, and identify infertility-related markers, molecular mechanisms, and epidemiology that may predict future neurodegenerative risk. By providing an up-to-date synthesis of the literature, the current review aims to inform clinical practice, guide future mechanistic investigations, and explore male infertility as a potential early-life risk indicator for NDDs.

## 2. Methodology

The Preferred Reporting Items for Systematic Reviews and Meta-Analyses (PRISMA) 2020 guidelines were used to inform the methodology of this review [15], which served as a checklist for a standardized, evidence-guided systematic review (see Supplementary Material S2).

### 2.1. Research Objectives

The primary aim of this systematic review is to capture and examine all relationships (e.g., associations, risks, and shared mechanisms) between human male infertility (which also included in vitro studies) and NDDs as reported in the published literature. Specifically, we examined potential associations, risks, and mechanisms of NDDs and male infertility (including diagnosed infertility, subfertility, or semen-based parameters) with a focus on molecular pathways. Altogether, these objectives helped summarize the reported link between male infertility and neurodegenerative diseases. This review’s objectives were guided and structured with a Population, Exposure, Comparator/Context, Outcome, and Study Design (PECOS) framework as shown in Box 1. Due to the high heterogeneity and limited number of captured studies, our initial secondary objectives outlined in the protocol on directionality were not feasible.

Box 1 **The PECOS framework used for the inclusion/exclusion of studies during the title-and-abstract and full-text screening.**

**Population**

Does the study involve human male participants? YES/NO

*If NO exclude*

In mixed-sex populations, can male-specific data be extracted? YES/NO

*If NO exclude*

**Exposure**

Study involved men with infertility/subfertility (as reported by study authors)? YES/NO

*If NO exclude*

**Context**

Does the study evaluate neurodegenerative diseases (e.g., Alzheimer’s, Huntington’s, Parkinson’s, etc.)? YES/NO

*If NO exclude*

**Outcome**

Does this study include associations/links between male infertility and neurodegenerative diseases? YES/NO

*If NO exclude*

**Study Design**

Is this study an empirical and/or original research? YES/NO

*If NO exclude*

### 2.2. Search Strategy

**Figure 1:**
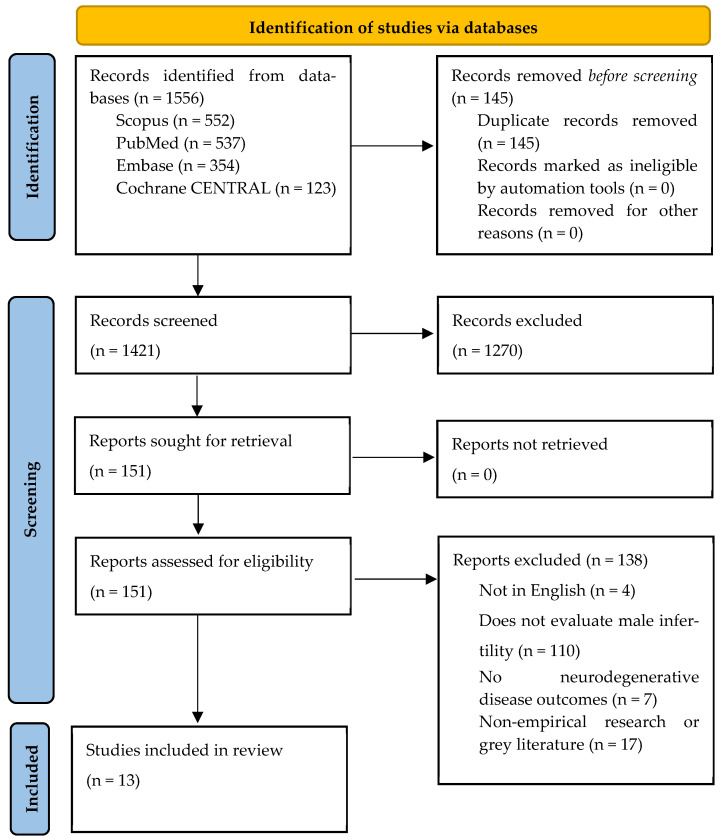
A PRISMA flow chart detailing the inclusion and exclusion of studies from the identified 1566 studies [15].

This systematic review searched four major electronic databases (PubMed, Embase, Scopus, and Cochrane CENTRAL) on 24 January 2026, for empirical and original English-language publications without date restrictions. The search strategy was developed by two reviewers (MA, NJ) and used Medical Subject Headings (MeSH) terms and Boolean AND/OR operators for advanced searching to optimize result yields. Accounting for differences in database search engine indexing, we adapted search strategies tailored to each database to improve sensitivity and specificity. Keywords (and their variations) that were used for each search strategy included “Male, Infertility, Semen, Sperm, Hypogonadism, Testosterone, Neurodegenerative Diseases, Alzheimer’s, Parkinson’s, Amyotrophic Lateral Sclerosis, Huntington, and Dementia”. The study selection process is illustrated in Figure 1. All search strategies for each database are documented in our registered protocol on PROSPERO, a public systematic review protocol database, to demonstrate transparency throughout all stages of this review (PROSPERO registration number: CRD420261301509).

### 2.3. Study Selection Process

The study selection process was conducted using Covidence (https://www.covidence.org; Veritas Health Innovation; Melbourne, Australia; accessed on 1 January 2026), an online software platform for managing reviews. Covidence was used for title and abstract screening, full-text review, blinding of reviewers, and reporting our results in accordance with the PRISMA 2020 guidelines [15]. Covidence also automatically removed uploaded duplicates and generated a PRISMA flow diagram to enhance workflow transparency.

A detailed inclusion/exclusion rubric (see Box 1) was agreed upon by all reviewers (MA, NJ, RK) before initiating the study selection process. The study selection process involved two stages. The first stage involved a title-and-abstract screening, and the second stage involved a more detailed review of the full-text of the articles that passed the title-and-abstract screening stage. Articles that entered either screening phase must receive two “Yes” votes from two independent reviewers (MA, NJ), who are blinded to each other’s vote, or one “Yes” and one “Maybe” vote before proceeding to the next stage. Articles that received two “No” votes were excluded at the respective stage, and any other voting combination was placed into a “Conflict” section where a third independent and blinded reviewer (RK) made a final decision.

### 2.4. Data Extraction

After completing the full-text screening of the articles, all included studies went through a data extraction phase where reviewers (SD, SK, NJ) collected the data via a standard data extraction form using the Microsoft Excel software in a manner designed as a PECOS table to uniformly collect the necessary data (see Box 2). Studies included were very heterogenous, with missing data due to differing study designs. Fields that were not applicable to a given study design (e.g., cytokine panels, epigenetic markers, and mitochondrial functionality assessments in purely epidemiological studies) were recorded as ‘not applicable’ rather than ‘missing data’. Missing data were acknowledged and transparently reported for each outcome type (see Table 1 and Table 2) and a risk-of-bias assessment was performed for each included study to assess bias risks from missing data. Following independent extraction, the extracted data was then verified by an additional blinded reviewer (NJ or MA) who had not extracted data initially from a particular study. Any controversies were resolved via group discussion. Extracted data for each included study characteristics, population demographics, exposure and comparator definitions, outcome measures (including NDDs and in vitro outcomes where applicable), effect estimates, and authors’ final conclusions, using the standardized extraction form. Data extraction specifically accounted for study setting (single versus multi-center and clinic versus population-based), and all effect estimates (odds-ratio, hazard-risk and relative-risk) were recorded with their respective 95% confidence intervals and *p*-values where available.

Box 2 **The extraction form used to capture the data of each included study (n = 13).**

**Study Details**

Digital Object Identified (DOI)

Author

Year of Publication

Journal

Country

Study Design

Data Source

Study Setting

**Population**

Sample Size

Age

Demographics

Comorbidities

Follow-up Duration

**Exposure**

Study Infertility Definition

Infertility Subtype/Etiology

Semen Parameters

Measured Reproductive Hormones

Timing and Assay Method

Use of Assisted Reproductive Treatment or Infertility Treatment

**Comparator** (if mentioned)

Comparator Definition

Matching Strategy

Confounders

**Neurodegenerative Disease Outcome**

Neurodegenerative Disease Type(s)

Outcome Ascertainment Method

Age at Diagnosis/Onset

Incidence/Prevalence

Severity/Progression Markers

Neurodegenerative Disease-Related Mortality

**In Vitro Study Outcomes**

Variant/Mutation

Epigenetic Markers

Transcriptomic Profile

Oxidative Stress Markers

Inflammatory Cytokine Panel

Immune-Mediated Outcomes

Mitochondrial Functionality

Biological Sample Source

Proteopathic Markers

**Effect Estimates**

Reported Measures

Final Conclusion

### 2.5. Data Synthesis

Quantitative meta-analysis was not feasible due to heterogeneity in study designs, exposure definitions, and outcome measures. Instead, we utilized a narrative descriptive synthesis approach to report our findings and explore the associations between NDDs and male infertility. A preliminary descriptive synthesis plan was described in our protocol, and reviewers (MA, NJ) built-upon the prospectively designed scaffold as they progressed through the screening stages whilst noting general concepts among included articles, developing a preliminary outcome section built upon the PECOS table in Box 2. Following data extraction, the reviewers (NJ, MA, SD, SK) met and discussed the findings in an iterative manner that helped define and refine our outcomes, generating overarching domains with subdomains that examined NDDs with male infertility. The synthesis reported our findings objectively via tables while using vivid examples to link NDDs with male infertility.

### 2.6. Quality Assessment

This review critically appraised all included studies. We used the Risk of Bias in Non-Randomized Studies of Exposures (ROBINS-E) tool for five observational studies [26]. This framework enabled us to evaluate studies across domains, including bias from confounding variables, bias in measurement of the exposure, bias in selection of participants into the study or the analysis, post-exposure interventions, missing data, outcome measures, and selective reporting. Additionally, we employed the office of health assessment and translational (OHAT) risk of bias tool to evaluate the quality of eight experimental and in vitro studies included in this review [27]. The OHAT tool assesses methodological quality and reporting standards of in vitro studies. The tool evaluates randomization, allocation concealment, participant selection, confounding, experimental conditions, blinding during study period, incomplete data, exposure characterization, outcome assessment, reporting, and other (including statistical analysis, adherence to study protocol, and accounting for unintended co-exposures). This dual-tool approach allowed us to utilize the appropriate quality assessment tools to assess risks of bias according to the study design, helping strengthen our interpretation of the molecular findings linking male infertility and NDD pathways. We also recorded the sources of funding, reported conflicts of interest, and author-reported limitations. These tools allow us to examine methodological coherence across the review and evaluate potential risks of bias that may impact effect measures. Final quality assessment scores (ROBINS-E: low, some concerns, high, or very high risk of bias; OHAT: probably low, definitely low, probably high, or definitely high) were determined for each study and presented in figure and table format, with further study-specific rationale and justification for each criteria presented in Supplementary Material S1.

## 3. Results and Discussion

### 3.1. Literature Search Results

The search strategy yielded 1566 studies across four databases (PubMed, Scopus, Embase, and Cochrane CENTRAL). Covidence automatically removed 142 studies as they were duplicates, while reviewers (MA, NJ) identified an additional three duplicates, bringing the total number of studies excluded before screening to 145. The authors conducted title and abstract screening for 1421 studies, of which 1270 were excluded at this stage due to exclusion criteria represented in Box 1. All 151 studies that entered the full-text review were successfully retrieved for the full-text. At this stage, 4 studies were excluded for unavailability in the English language, 110 studies were excluded since they did not evaluate male infertility, 7 studies were excluded as they did not mention NDDs, and 17 studies were excluded as they were non-empirical or gray literature (e.g., non-peer reviewed publications or posters). Thirteen studies were deemed to match the study inclusion criteria (see Box 1) and incorporated as the body of evidence for the current systematic review.

### 3.2. Study Characteristics

The 13 studies included in this review were published between 1995 and 2025 and addressed the links, associations, and mechanisms between male infertility and NDD. The included studies were conducted in China (3 studies) [16,18,23], Portugal (3 studies) [12,17,21], the United States (3 studies) [19,20,24], Taiwan (1 study) [14], Ireland (1 study) [22], Grenada (1 study) [20], Saudi Arabia (1 study) [20], India (1 study) [13], and Canada (1 study) [25] representing a wide geographical distribution. Infertility definitions ranged from WHO-based semen thresholds in genomic studies to reproductive outcome proxies in epidemiological studies, representing a significant source of heterogeneity as reported in Table 1. These studies examined molecular features of male fertility relevant to mitochondrial bioenergetics, oxidative stress, DNA integrity, and protein pathways, and some also reported direct epidemiological risk estimates. Table 1 represents study characteristics for all included studies.

### 3.3. Evidence Outcomes

Across the 13 included studies, associations between NDD and male infertility were categorized into domains and subdomains (Table 2) guided by identified patterns described by the included studies. The domains were further categorized into four sections that formed our observations seen in the below discussion: mitochondria and bioenergetics, oxidative stress and DNA integrity, proteostatic and proteopathic pathways, and regulatory mechanisms.

### 3.4. Quality Appraisal

**Figure 2:**
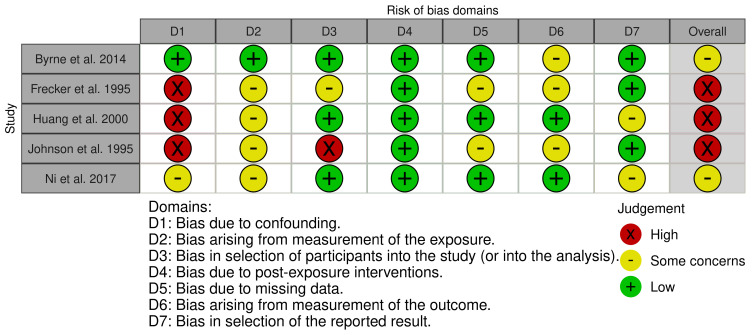
ROBINS-E traffic-light plot for included studies (n = 5) [14,18,22,24,25].

Quality appraisal was conducted using the ROBINS-E tool for the five observational and population-based studies and the OHAT tool for the eight experimental and in silico investigations. Of those using ROBINS-E, none demonstrated an overall low risk of bias, two had some concerns, and three were classified as having high risk of bias. A detailed risk of bias assessment for each study is presented in Figure 2 and Table 3, respectively. Within this framework, a low risk of bias indicates a well-executed study with reliable results, whereas some concerns indicate important limitations that may influence the findings but do not severely undermine their validity. A high risk of bias points to significant methodological flaws that could substantially affect the estimated effect and reduce confidence in the outcomes.

For the experimental studies evaluated via OHAT, a definitely low risk indicates direct evidence of practices that effectively minimize bias, while a probably low risk suggests indirect evidence or deviations unlikely to impact results. Conversely, a probably high risk reflects insufficient reporting or indirect evidence of bias, and a definitely high risk signifies direct evidence of significant methodological flaws likely to compromise the findings. Based on these criteria, two studies [19,23] were classified as having an overall low risk of bias, five as probably high risk [12,13,17,20,21], and one as definitely high risk [16].

Analysis of the ROBINS-E assessments indicates that Domain 1 (Bias due to confounding) was the most frequent source of concern across the literature. Many studies, particularly genomic and clinical case-control analyses such as those by Huang et al. [14] and Ni et al. [18], faced significant challenges in controlling for lifestyle factors, age, and environmental exposures that independently influence both mitochondrial health and reproductive outcomes. In contrast, Domain 4 (Bias due to post-exposure interventions) consistently showed the lowest risk because most studies were observational or followed standardized protocols, resulting in minimal experimental drift.

Similarly, the OHAT appraisals identified Blinding During Study and Outcome Assessment as the most problematic areas, as information regarding these domains was frequently not reported (NR) or demonstrated a high risk of bias. This may be due to differences in reporting guidelines for in vitro and molecular experiments, in which biochemical assays are often perceived as inherently objective. However, the potential for subconscious operator bias remains a limitation. In contrast, domains relating to Experimental Conditions, Exposure Characterization, and Reporting consistently showed a definitely low risk, reflecting high technical reliability and transparency in the experimental environment. While Domain 7 (Bias in selection of the reported result) of the ROBINS-E tool presented occasional risks, the high technical objectivity in Domain 6 (Bias in measurement of outcomes), supported by automated sequencing and mass spectrometry, ensured that the risk of observer subjectivity remained low once data was collected.

#### 3.4.1. Principal Findings

Despite heterogeneity on the exact clinical ‘risk’ between infertility and NDD, some studies suggest potential molecular overlap between the two. Preliminary evidences suggests that both infertility and NDDs may involve broadly similar cellular stress pathways, including mitochondrial dysfunction, oxidative/genotoxic stress, and proteostasis imbalance [12,14,16,17,18,21,23]. At a cellular level, both sperm and neurons share similar microenvironment requirements, including extreme adenosine triphosphate (ATP) dependence and redox homeostasis with failure of antioxidant mechanisms [14,17,18,21]. The shared long-range compartmentalized architecture of these two post-mitotic cells may potentially explain a heightened reliance on ATP and, consequently, how disturbances in proteostasis and mitochondrial function may serve as shared-vulnerability nodes [14,17,18,21]. This shared vulnerability may likely be rooted in that both neurons and spermatozoa are specialized terminal cells with finite regenerative capacity; thus, their seemingly shared molecular mechanisms could suggest that a sub-clinical defect in sperm might represent a premature signal for a lower systemic threshold of neurodegeneration later in life. In the following sections, findings from the 13 included studies are presented alongside supporting background evidence from the broader literature, which is cited separately to maintain clarity regarding the evidence base.

#### 3.4.2. Mitochondrial Genome Integrity and Bioenergetics: A Direct Bridge to Motility Failure

Mitochondrial DNA (mtDNA) damage and the resulting energy failure have been reported in both sperm and neurons. Huang et al. identified that the enrichment of the mtDNA 4977 base pairs (bp) ‘common deletion’ leads to the loss of genes encoding subunits of the respiratory chain, Complexes I, IV, and V, which are significantly associated with low sperm motility (*p* < 0.01). This inverse correlation suggests that sperm motility may serve as a functional indicator of respiratory competence [14]. Spermatozoa may be uniquely sensitive to such deletions potentially due to lower mitochondrial counts, meaning that even smaller-scale mtDNA damage may lead to mitochondrial and, therefore, spermatic dysfunction [25]. As such deletions are often observed in aging neurons, with further research needed to determine if detecting them in the spermatozoa of younger men could serve as a potential predictor for early-onset NDD.

Beyond common deletions, specific point variants also appear to play a role. Ni et al. identified mtDNA variants in genes MT-ND4 (g.11084A>G) and MT-TL1 (g.3263C>T) as significant risk factors in the development of asthenozoospermia (*p* < 0.05). This supports the hypothesis that energy failure may be encoded into the mitochondrial genome, perhaps forming a potential genetic/energetic axis for motility impairment [18]. While mitochondrial dysfunction is a central mechanism in multiple NDDs, spermatozoa are more readily accessible for routine clinical evaluation than neurons, potentially providing preliminary insights into a patient’s systemic mitochondrial health [28,29,30]. The broader NDD literature mirrors this genetic/energetic axis, recognizing that mtDNA variants that are not traditionally ‘pathogenic’ in a Mendelian sense are now recognized as potential modifiers of disease onset and severity. Chinnery et al. demonstrated that mtDNA variants associated with haplogroups H, J, Uk, and T may influence the susceptibility and expressivity of NDDs such as AD and PD [28]. Given the heavy reliance of spermatozoa on ATP for motility (similar to the energy requirements of neurons) their mtDNA failures in infertility cases may reflect a broader, systemic mitochondrial vulnerability [14,17,18,21].

#### 3.4.3. Oxidative Stress and DNA Integrity: Upstream ‘Common Soil’

Sperm-NDD network and proteome overlap analyses have revealed that oxidative stress may act as a mechanistic feeder, potentially triggering a genotoxic cycle that both spermatozoa and neurons may be vulnerable to. In these models, oxidative stress was observed to precipitate DNA damage, which may subsequently worsen mitochondrial function and lead to the failure of proteostasis systems. This could align certain infertility phenotypes with molecular trajectories reminiscent of those seen in NDDs [14,17,18,21]. This ‘common ground’ observation is supported by evidence that systemic oxidative indices, such as Urinary 8-hydroxy-2′-deoxyguanosine (8-OHdG), a marker of global DNA oxidation, may correlate with both sperm DNA fragmentation and cognitive decline in aging populations [29,30]. Given that spermatozoa possess limited DNA repair capacity once they leave the testes, they may be the first cells to manifest the effects of this systemic oxidative feeder mechanism. This can, in theory, position them as potentially sensitive, early biomarkers of the same oxidative stress that also drives neurodegeneration [31,32].

#### 3.4.4. Proteostasis and Proteopathic Proteins: Amyloid Precursor Protein/Amyloid and α-Synuclein Signals in Reproductive Biology

Several included studies noted a role for canonical NDD-associated proteins and protein-handling pathways within sperm/testis biology, indicating a possible cross-tissue relevance of proteostasis modules [12,17,21,23]. Fardilha et al. localized Amyloid Precursor Protein (APP)-family proteins (recognized by the 6E10+ antibody) specifically to the sperm tail and head surface, while Amyloid Precursor-like Protein 2 (APLP2) was found in the mid-piece and equatorial region [12]. It is perhaps plausible that these cell types share certain folding or processing features, particularly as Yang et al. achieved the high-resolution crystallographic characterization of an APP-family-related extracellular fragment (YWK-II) extracted from the sperm membrane [23].

As APP and its homologues are localized in human sperm, it has been proposed that their dysfunction may contribute to reduced fertility. Tavares et al. demonstrated that the experimental exposure of human sperm to Aβ1-42 oligomers significantly decreased motility and viability (*p* < 0.05) while inducing a rise in intracellular Ca^2+^ and impairing acrosomal integrity [17]. Furthermore, in silico modeling by Krishnan et al. identified 12 overlapping molecules between α-synuclein (SNCA) and infertility conditions, predicting that SNCA activation may exacerbate both NDD and reproductive signaling via dihydrotestosterone (DHT) and caspases [19].

The relevance of α-synuclein to the male reproductive tract likely stems from its physiological role in membrane remodeling and vesicle trafficking, as noted by Schechter et al., processes that are vital to both acrosome reaction in sperm and neurotransmitter release in the brain [31]. By viewing α-synuclein and its related proteins as shared regulators of vesicular dynamics, these findings suggest that proteopathic stressors affecting key neurodegenerative pathways might also impact the function of spermatozoa [32].

#### 3.4.5. Regulatory Layers: miRNAs, Age-Linked Proteome Remodeling, and Pathway-Level Overlap

Emerging multi-omics evidence hints at a possible convergence of molecular programs between spermatogenic failure and neurodegeneration, largely centered on mitochondrial integrity and protein homeostasis. At a post-translational level, Tang et al. found that testicular miRNA dysregulation in NOA involved 449 differentially expressed miRNAs, with validated targets including genes associated with Parkinson’s, Huntington’s, and Alzheimer’s diseases (e.g., ATP5G2, COX6C, SDHD) [16]. The highlighted miRNA clusters are potentially significant as miRNAs are being investigated as “liquid biopsies” for early NDD detection [32]. The presence of similar regulatory signatures (miRNA clusters in dysfunctional testicular tissue) suggests a possible systemic, rather than tissue-specific, vulnerability.

This potential overlap extends to the proteome. Beg et al. identified 588 sperm proteins and observed a significant increase in NDD-related pathways (*p* < 0.05) in the aging sperm proteome [17]. This potentially suggests an age-associated accumulation of cellular stress and compensatory proteostasis activation. This was further supported by Silva et al. through systems-level integration, revealing the overlap of 21 proteins shared between the spermatozoa proteome and four major NDDs (AD, Parkinson’s Disease [PD], Huntington’s Disease [HD], and Amyotrophic Lateral Sclerosis [ALS]) primarily involving protein folding chaperones and the ubiquitin-proteasome system [21]. Such findings necessitate further research to map the potential overlap between mitochondrial and proteostasis-related networks in both male reproductive and neural tissue pathways.

#### 3.4.6. Cross-Disease Heterogeneity in “Risk” and Reproductive Outcomes

Direct epidemiologic signals linking reproductive history to neurodegeneration are limited and heterogenous across disorders, as reported in pedigree/family-based analyses [20,22,24]. In AD, family-based analyses indicate that higher infertility prevalence is clustered specifically in the sporadic AD subtype (40%) compared to familial AD (21%). Interestingly, this study also noted an association with HLA-DR3 and a clustering of autoimmune conditions like asthma [22].

By contrast, Byrne et al. observed that ALS patients had significantly more children than controls (*p* < 0.001), suggesting increased fecundity in some contexts [20]. However, pedigree analysis by Johnson et al. revealed that specifically male ALS-gene carriers exhibited significantly reduced fecundity (2.42 vs. 3.25 children/parent) decades before neurological symptoms appeared, a pattern notably absent in PD gene-carriers [24]. This paradox that genetically predisposed individuals (particularly ALS) may exhibit higher fecundity and may be explained through several potentially plausible mechanisms derived from antagonistic pleiotropy. Certain genetic traits may favor early-life reproductive success but may increase disease risk in later life [32]. Androgen receptor CAG repeat polymorphisms have linked reproductive fitness to neurodegeneration, where shorter repeats could confer higher androgen receptor sensitivity and transcriptional activity [33]. However, shorter repeats have also been linked to increased Alzheimer’s disease risk, particularly in early-onset cases [34]. ALS-susceptibility genes such as C9orf72, SOD1, TARDBP, and FUS converge on pathways involving impaired RNA metabolism, altered proteostasis, cytoskeletal defects, and mitochondrial dysfunction that manifest primarily in later life [35]. Notably, SOD1-null mice demonstrate impaired spermatogenesis with increased oxidative stress and DNA damage, suggesting that the complete loss of SOD1 function may be detrimental to male fertility; however, certain SOD1 and SOD2 gene variants show pleiotropic effects, with some polymorphisms associated with reduced male infertility risk, suggesting complex gene-environment interactions. This pattern of fecundity demonstrates heterogeneity in the relationship between genotypes of neurodegeneration and infertility. The above evidence may support prioritizing infertile males with non-genetic NDD in future research; thus, reducing pleiotropic confounding between reproductive dysfunction and neurodegeneration. To further maximize mechanistic insights and ensure increased generalizability, further research can be stratified by genetic carrier status to include both familial and sporadic NDD cohorts. Overall, while a mechanistic convergence on mitochondrial, redox, and proteostasis pathways may plausibly link distinct infertility phenotypes to NDD, the current lack of homogeneity across studies necessitates caution in generalizing these risks.

#### 3.4.7. Limitations

While this systematic review provides a comprehensive synthesis of the molecular links between male infertility and NDDs, several limitations must be acknowledged. First, the included evidence base is limited by significant heterogeneity in study design. Of the thirteen included studies, four were case-control studies (one of which was population-based), six were experimental investigations (including two in vitro studies), two were bioinformatics network analyses, and one was a retrospective pedigree study. This variability, coupled with inconsistent definitions of both infertility and NDD outcomes, precluded any quantitative meta-analysis and necessitated a narrative interpretation of the findings. Secondly, ten of the thirteen included studies were assessed as having a moderate to high risk. Many studies did not adequately control key confounding variables, such as age, lifestyle factors, and environmental exposures, which could independently influence both reproductive and neurological health. This limitation is further exacerbated by the significant disparity in study scale across the evidence base, ranging from high-powered population-based registries with over 700 participants to highly specialized molecular investigations with sample sizes as small as six individuals. As these studies do not carry equal statistical weight, the findings from smaller, exploratory analyses primarily offer mechanistic plausibility rather than the robust generalizability of larger cohort designs. Finally, the cross-sectional nature of most molecular studies limits the ability to infer causality; although they establish strong associations and mechanistic plausibility, they cannot determine whether male infertility is a direct cause, a consequence, or simply a parallel manifestation of an underlying systemic pathology that also leads to NDDs. It therefore remains unclear whether the molecular overlap identified in this review reflects a specific biological link between male reproductive and neurological health, or a broader shared vulnerability to systemic aging and cellular stress. Future studies should include comparison groups with other chronic conditions to test the specificity of these associations.

#### 3.4.8. Future Directions and Implications

This integrative perspective reinforces the concept that male reproductive health serves as an early indicator of broader systemic and neurological risk. These findings support the emerging paradigm that reproductive health parameters serve as accessible early-life indicators of systemic biological integrity, with implications extending beyond fertility to neurological and general health outcomes. For example, emerging evidence that post-translational modifications in spermatozoa, such as poly(ADP-ribosyl)ation, may serve as early molecular biomarkers of sperm health and, by extension, systemic biological status [36]. In future explorations of the relationship of male infertility to health parameters, more high-quality research, such as longitudinal cohort studies linking fertility parameters with registry-based NDD outcomes, can be done to determine actual NDD incidence rates. Current evidence relies heavily on cross-sectional molecular studies and limited family-based analyses. Such studies can be supplemented with randomized controlled trials testing whether antioxidant therapy or mitochondrial-targeted interventions to investigate the utility of preventive interventions.

Further molecular analysis such as phenotype-stratified cohorts (e.g., NOA vs. asthenozoospermia vs. idiopathic infertility) can be done to understand why some mitochondrial variants cause primarily reproductive phenotypes while others affect the nervous tissue. Combining standardized semen metrics plus deep molecular profiling of mitochondria, DNA integrity and proteastasis could reveal tissue-specific therapeutic targets. Additionally, multi-omics integration (including miRNA profiling) across matched samples of sperm, blood and CSF (when available) would comprehensively map the molecular landscape linking these conditions. There must be particular focus on age-dependent changes, as both spermatogenesis and neurodegeneration show strong age associations [37,38].

Reframing male infertility evaluation as a health surveillance opportunity may offer a unique perspective in screening for neurodegenerative diseases. Risk stratification algorithms integrating reproductive parameters with other early-life markers may serve as an entry point in fertility clinics to identify high-risk individuals for targeted surveillance. Semen quality demonstrates dose-response relationships with mortality [39]; therefore, if similar gradients exist for neurodegenerative risk, standardized thresholds could trigger enhanced monitoring protocols including cognitive screening, neuroimaging biomarkers, or CSF analysis in young men with severe reproductive function. These directions represent speculative, hypothesis-driven proposals. Validation through large-scale prospective studies is essential before male infertility assessment can be considered as a screening tool for NDD risk.

## 4. Conclusions

This systematic review may indicate a potential molecular convergence between male infertility and NDD pathways, suggesting that male infertility may represent a peripheral readout of shared molecular vulnerabilities. The primary mechanisms suggesting the bridging of these conditions include mitochondrial dysfunction and bioenergetic failure, shared susceptibility to oxidative stress and impaired DNA integrity, and proteostasis imbalances. However, the current evidence is preliminary and largely mechanistic; whether these associations translate into clinically meaningful neurodegeneration risk prediction requires validation through large-scale, prospective longitudinal cohort studies.

## Figures and Tables

**Table 1:** Study characteristics of included studies (n = 13).

Study (Author)	Year	Country	Design	Sample Size	Infertility Phenotype	NDD Type (s)	Biospecimen/Sample	NDD-Linked Markers	Key Outcomes/Effect Estimates (as Extracted)
Tang et al. [16]	2018	China	Experimental (Cross-sectional comparison using microarray analysis and qRT-PCR validation)	6 men	Non-obstructive azoospermia (NOA) following bilateral cryptorchidism	Parkinson’s disease, Huntington’s disease, and Alzheimer’s disease	Testicular tissue (biopsy)	Target genes associated with Parkinson’s disease (ATP5G2, COX6C, SDHD, UBB, UBE2L6, COX7A2L, GNAL, COX8C) and Alzheimer’s disease (ATP5G2, COX6C, CASP12, SDHD, PPP3CC, COX7A2L, LPL, COX8C)	449 miRNAs significantly differentially expressed: 9 of 12 selected miRNAs validated by qRT-PCR (*p* < 0.05). miRNA details: hsa-miR-449a (Downregulated) hsa-miR-34c-5p (Downregulated) hsa-miR-512-5p (Downregulated) hsa-miR-182-5p (Downregulated) hsa-miR-138-5p (Downregulated) hsa-miR-299-5p (Upregulated) hsa-miR-199a-3p (Upregulated) hsa-miR-142-3p (Upregulated) hsa-miR-409-3p (Upregulated)
Tavares et al. [17]	2017	Portugal	Experimental (In vitro study)	7 men	Healthy donors (Normal sperm parameters)	Alzheimer’s Disease (Aβ1-42 peptide)	Human sperm	Amyloid-beta 1-42	Aβ1-42 decreased motility (*p* < 0.05) and viability (*p* < 0.05). Reduced acrosomal integrity (*p* < 0.05). Significant rise in intracellular calcium ([Ca^2+^]) at 20 μM (*p* < 0.05).
Ni et al. [18]	2017	China	Case-control analysis (Genomic study)	177 subjects (97 infertile patients, 80 fertile controls)	Asthenozoospermia	Mitochondrial Neurodegenerative Disorders (specifically LHON and MELAS-related pathways via MT-ND4 and MT-TL1 genes)	Human sperm	Mutations in subunits of NADH dehydrogenase (Complex I) and mitochondrial tRNA	Mutation g.11084A>G (MT-ND4) and g.3263C>T (MT-TL1) identified as significant risk factors (*p* < 0.05)
Krishnan et al. [19]	2023	USA	Bioinformatic network analysis (In silico study)	Not relevant (Bioinformatic database analysis)	Not specified	Parkinson’s Disease	In silico (Database-derived)	Alpha-synuclein (SNCA)	12 overlapping molecules; MAP predicted SNCA activation exacerbates PD and IC via DHT and caspases.
Huang et al. [14]	2000	Taiwan	Case-control	62 (25 normal motility, 37 abnormal motility)	Asthenozoospermia	Mitochondrial Neurodegenerative Disorders (specifically Mitochondrial Myopathies where the 4977 bp “common deletion” is a hallmark)	Human sperm	Truncated or missing subunits of NADH dehydrogenase and ATP synthase.	Significant difference in deletion frequency (*p* < 0.01); significant inverse correlation between deletion load and motility.
Beg et al. [20]	2025	Saudia Arabia, USA, Grenada	Experimental	Divided into three age groups (Total identified proteins: 588)	Age-related fertility decline	General Neurodegenerative Diseases (Pathways associated with NDDs identified via enrichment analysis)	Human sperm	Protein folding pathways significantly increased with age (associated with NDD risk)	588 proteins identified; significant increase in NDD-related pathways (*p* < 0.05); decrease in flagellated motility
Silva et al. [21]	2015	Portugal	Bioinformatic network analysis (Systems biology integration)	1076 proteins (human sperm proteome)	General Male Infertility	Alzheimer’s Disease (AD), Parkinson’s Disease (PD), Huntington’s Disease (HD), Amyotrophic Lateral Sclerosis (ALS)	Human sperm	Ubiquitin-proteasome system components and protein folding chaperones	Identified 21 proteins shared between the sperm proteome and all four neurodegenerative diseases studied.
Byrne et al. [22]	2014	Ireland	Population-based, case-control study	747 (511 patients with ALS, 236 healthy controls)	Not relevant	Amyotrophic Lateral Sclerosis (ALS)	Blood/DNA (for C9orf72 analysis) and demographic records	C9orf72 protein expansion	ALS patients had significantly more children than controls (*p* < 0.001); no difference found in number of siblings (*p* = 0.44)
Yang et al. [23]	2003	China	Experimental (in vitro)	Not relevant (recombinant protein crystals)	Not mentioned	Alzheimer’s Disease (YWK-II/APPH is a homolog of the βA4-amyloid precursor protein (APP))1β42 peptide)	Recombinant Protein: Expressed in *Escherichia coli* (strain BL21). Original Protein Context: Human sperm membrane.	YWK-II is a member of the amyloid precursor protein (APP) family	Crystal diffraction resolution (2.9 Å for native; 2.38 Å for SeMet derivative). Space group (P21). Unit-cell parameters (a = 46.0, b = 43.7, c = 90.2 Å)
Johnson et al. [24]	1995	USA	Retrospective family study/pedigree analysis	3968 individuals (ALS: 89 kindreds containing 1619 individuals (412 affected)) (Parkinson Disease: 214 kindreds containing 2349 individuals (613 affected))	Not mentioned	Amyotrophic Lateral Sclerosis (ALS) and Parkinson’s Disease	Pedigree analysis only	Not mentioned	Number of children per gene-carrier parent (male vs. female) and proportion of childless gene-carriers
Fardilha et al. [12]	2007	Portugal	Experimental	Not mentioned	Not relevant	Alzheimer’s Disease (AD)	Human sperm and testis	Amyloid Precursor Protein (APP): “Bona fide” APP in human sperm (specifically recognized by 6E10 antibody). Amyloid Precursor-Like Protein 2 (APLP2)	APP (6E10+): Localized mainly to the sperm tail (main piece) and head surface. APLP2 (KPI+): Localized mainly to the mid-piece and equatorial region of the head.
Vijayalaxmi et al. [13]	2005	India	Experimental	Not relevant	Idiopathic (lack of FASA expression)	Huntington’s Disease (HD)	Human epididymal library, mouse testis/germ cells	Huntingtin Protein (HDP)	Sequence homology (87% to HDP). Protein structure: 2 N-glycosylation sites, 4 O-glycosylation sites, 22 phosphorylation sites. Hydrophobicity: FASA is a hydrophobic protein, likely associated with membrane phospholipids.
Frecker et al. [25]	1995	Canada	Case-Control	100 Alzheimer patients and their 1884 relatives Group 1: Familial Dementia of the Alzheimer Type (FDAT) (n = 47). Group 2: Non-Familial Dementia of the Alzheimer Type (NFDAT) (n = 53).	Not clinically diagnosed; defined by reproductive outcome (childlessness)	Alzheimer’s Disease (AD) (divided into Familial and Sporadic/Non-Familial subtypes).	Human blood		Infertility Prevalence: Significantly higher in NFDAT relatives (21 families, 40%) compared to FDAT relatives (10 families, 21%) (OR = 2.43). Sex Difference in Infertility: Among infertile relatives, males comprised a larger proportion in the NFDAT group (42%) compared to the FDAT group (20%).

**Table 2:** Evidence map: domains, subdomains, extracted outcomes, and references.

Domain	Subdomain	Outcomes/Key Findings	Reference (s)
Clinical associations and reproductive fitness	Infertility prevalence in AD families (familial vs. non-familial)	Infertility prevalence was higher in non-familial dementia of the Alzheimer type (NFDAT) relatives (21 families, 40%) than familial dementia of the Alzheimer type (FDAT) relatives (10 families, 21%) (OR = 2.43). Sex difference: among infertile relatives, males comprised a larger proportion in NFDAT (42%) vs. FDAT (20%).	Frecker et al. (1995) [25]
	Fertility outcomes in ALS cohorts	ALS patients had significantly more children than controls (*p* < 0.001), with no difference in number of siblings (*p* = 0.44).	Byrne et al. (2014) [22]
	Reproductive patterns in familial ALS/PD gene-carriers	In pedigree-based analysis defining “reduced fecundity” as fewer children per gene-carrier parent, male ALS gene-carriers showed significantly reduced fecundity vs. female carriers (2.42 vs. 3.25 children/parent), occurring decades before neurological symptoms and interpreted as a pleiotropic effect of ALS susceptibility on male fertility.	Johnson et al. (1995) [24]
Transcriptomic/epigenetic regulation	Testicular miRNA dysregulation with NDD-linked target genes	In post-cryptorchidopexy NOA testicular tissue, 449 miRNAs were significantly differentially expressed, and 9/12 candidate miRNAs were validated by qRT-PCR (*p* < 0.05). NDD-linked targets highlighted included Parkinson’s disease-associated genes (ATP5G2, COX6C, SDHD, UBB, UBE2L6, COX7A2L, GNAL, COX8C) and Alzheimer’s disease-associated genes (ATP5G2, COX6C, CASP12, SDHD, PPP3CC, COX7A2L, LPL, COX8C).	Tang et al. (2018) [16]
	Age-associated sperm proteome and PTM shifts	588 sperm proteins were identified across age strata, with significant enrichment of neurodegenerative disease–related pathways (*p* < 0.05) and a decrease in flagellated motility alongside age-associated changes in metabolism and protein folding.	Beg et al. (2025) [20]
Proteome/network overlap across tissues	Shared sperm proteins across major NDDs	Systems-biology integration of the human sperm proteome (1076 proteins) identified 21 proteins shared between the sperm proteome and datasets for AD, PD, HD, and ALS, highlighting pathway-level overlap involving protein folding and oxidative stress.	Silva et al. (2015) [21]
Proteostasis and proteopathic proteins	APP superfamily in sperm (localization)	APP immunoreactivity (6E10+) localized mainly to the sperm tail (main piece) and head surface, while APLP2 (KPI+) localized mainly to the mid-piece and equatorial region of the head, supporting presence of APP-family proteins in sperm with compartment-specific distribution.	Fardilha et al. (2007) [12]
	APP family structural biology (APP homolog fragment)	Crystallographic characterization of an extracellular fragment (X3) of YWK-II, a member of the amyloid precursor protein (APP) family, achieved diffraction resolution of 2.9 Å (native) and 2.38 Å (SeMet derivative) (structural evidence supporting APP-family protein study in relevant systems).	Yang et al. (2003) [23]
	Alpha-synuclein-centered infertility-PD network	In silico network analysis identified 12 overlapping molecules between α-synuclein (SNCA) and an infertility condition (IC), with modeling predicting SNCA activation as a regulator exacerbating PD- and infertility-related signaling via DHT and caspases (with cytokines noted as key nodes in the network).	Krishnan et al. (2023) [19]
Mitochondrial genome integrity and bioenergetics	mtDNA point mutations in asthenozoospermia	Two mtDNA variants were identified as significant risk factors in asthenozoospermia: g.11084A>G (MT-ND4) and g.3263C>T (MT-TL1) (*p* < 0.05).	Ni et al. (2017) [18]
	mtDNA “common deletion” (4977 bp) and sperm motility	The 4977 bp mtDNA deletion was significantly associated with low sperm motility, with a significant inverse correlation between deletion load and motility (*p* < 0.01). The deletion was noted to remove genes encoding subunits of respiratory chain Complexes I, IV, and V, implicating impaired bioenergetics.	Huang et al. (2000) [14]
Proteotoxic stress impacting sperm function	Amyloid-β exposure and sperm dysfunction	Aβ1-42 decreased sperm motility (*p* < 0.05) and viability (*p* < 0.05), reduced acrosomal integrity (*p* < 0.05), and induced a significant rise in intracellular Ca^2+^ at 20 μM (*p* < 0.05), supporting direct sperm dysfunction following amyloid exposure.	Tavares et al. (2017) [17]
Cross-tissue molecular homology	Testis proteins with homology to neurodegenerative proteins	Fertility-associated sperm antigen (FASA) showed 87% sequence homology to a huntingtin-related protein (HDP), with predicted PTM features (2 N-glycosylation sites, 4 O-glycosylation sites, 22 phosphorylation sites) and hydrophobicity consistent with membrane association, suggesting shared motifs between fertility-associated and neurodegeneration-linked proteins.	Vijayalaxmi et al. (2005) [13]
Immunogenetic and inflammatory links	HLA associations and autoimmunity clustering in AD pedigrees	HLA-DR3 was significantly associated with familial AD (OR = 3.78), and HLA-A2 was increased in non-familial AD females (OR = 6.60). Autoimmune diseases clustered significantly in non-familial AD patients (OR = 2.70) and their relatives (OR = 2.72), and asthma was more common in non-familial AD relatives (OR = 3.84).	Frecker et al. (1995) [25]

**Table 3:** OHAT summary for the included studies (n = 8).

Study	Randomization	Allocation Concealment	Participant Selection	Confounding	Experimental Conditions	Blinding During Study	Incomplete Data	Exposure Characterization	Outcome Assessment	Reporting	Other	Overall
Tang et al. 2018 [16]	N/A	N/A	+	-	++	NR	+	+	--	++	+	--
Tavares et al. 2017 [17]	++	++	N/A	N/A	++	NR	+	++	-	++	++	-
Krishnan et al. 2023 [19]	N/A	N/A	N/A	N/A	++	N/A	+	++	++	++	+	+
Beg et al. 2025 [20]	N/A	N/A	++	+	++	NR	+	++	+	++	+	-
Silva et al. 2015 [21]	N/A	N/A	N/A	N/A	++	NR	+	+	-	++	+	-
Yang et al. 2003 [23]	N/A	N/A	N/A	N/A	++	N/A	++	++	++	++	++	++
Fardilha et al. 2007 [12]	N/A	N/A	-	N/A	++	NR	+	++	-	++	+	-
Vijayalaxmi et al. 2005 [13]	N/A	N/A	-	N/A	++	NR	+	++	-	++	+	-

Office of Health Assessment and Translation (OHAT) Risk of Bias Tool [27]. Key: N/A: Not applicable, NR: Not reported, -: Probably low, --: Definitely low, +: Probably high, ++: Definitely High. Overall risk of bias key: 1. Probably low risk of bias: There is indirect evidence of low risk-of-bias practices OR it is deemed that deviations from low risk-of-bias practices for these criteria during the study would not appreciably bias results, including consideration of direction and magnitude of bias. 2. Definitely low risk of bias: There is direct evidence of low risk-of-bias practices (may include specific examples of relevant low risk-of-bias practices). 3. Probably high risk of bias: There is indirect evidence of high risk-of-bias practices OR there is insufficient information (e.g., not reported or “NR”) provided about relevant risk-of-bias practices. 4. Definitely high risk of bias: There is direct evidence of high risk-of-bias practices (may include specific examples of relevant high risk-of-bias practices).

## Data Availability

No new data were created or analyzed in this study. Data sharing is not applicable to this article.

## Supplementary Materials

The following supporting information can be downloaded at: https://www.mdpi.com/article/10.3390/ijms27073222/s1.

## Abbreviations

The following abbreviations are used in this manuscript:

AD	Alzheimer’s disease
ALS	Amyotrophic lateral sclerosis
APP	Amyloid precursor protein
ATP	Adenosine triphosphate
DHT	Dihydrotestosterone
HD	Huntington’s disease
miRNA	MicroRNA
mtDNA	Mitochondrial DNA
NDDs	Neurodegenerative diseases
NOA	Non-obstructive azoospermia
PD	Parkinson’s disease
PECOS	Population, Exposure, Comparator/Context, Outcome, and Study Design
PRISMA	Preferred Reporting Items for Systematic Reviews and Meta-Analyses
OHAT	Occupational Health Assessment and Translational
ROBINS-E	Risk of Bias in Non-Randomized Studies of Exposure
SNCA	Synuclein alpha (gene encoding alpha-synuclein)
